# A solitary perioral plaque mimicking an accentuated unilateral perioral fold

**DOI:** 10.1016/j.jdcr.2026.09.013

**Published:** 2026-09-15

**Authors:** Halim Jo, Ji Hyun Lee

**Affiliations:** a Department of Dermatology, Seoul St. Mary’s Hospital, College of Medicine, The Catholic University of Korea, Seoul, Republic of Korea; b Mitochondrial Immunomedicine Center for Global Health Therapeutics (MIGHT), College of Medicine, The Catholic University of Korea, Seocho-gu, Seoul, Republic of Korea

**Keywords:** hyperplasia, sebaceous gland diseases, sebaceous glands

## Case description

A 44-year-old man presented with an asymptomatic, slowly enlarging plaque on the left perioral skin that had developed over 2-3 years, creating the appearance of an accentuated unilateral perioral fold (Fig 1, *A*). The vermilion border was spared, and prior oral antibiotics were ineffective. He had a history of atopic dermatitis without immunosuppressant use. Examination revealed a solitary, well-demarcated, and skin-colored to brownish plaque with a smooth surface and preserved terminal hairs (Fig 1, *B*). Dermoscopy showed grouped whitish-yellow sebaceous lobules with peripheral crown vessels (Fig 2). Histopathologic examination revealed enlarged sebaceous lobules composed of mature sebocytes arranged around centrally dilated sebaceous ducts, some opening onto the epidermal surface (Fig 3). The peripheral basaloid germinative layer was inconspicuous, limited to 1 to 2 cell layers. The overlying epidermis was unremarkable, without papillomatosis or epidermal hyperplasia. Cytologic atypia and increased mitotic activity were absent, and there were no follicular malformations, ectopic apocrine glands, perifollicular fibrosis, or cystic structures.

**Fig 1:**
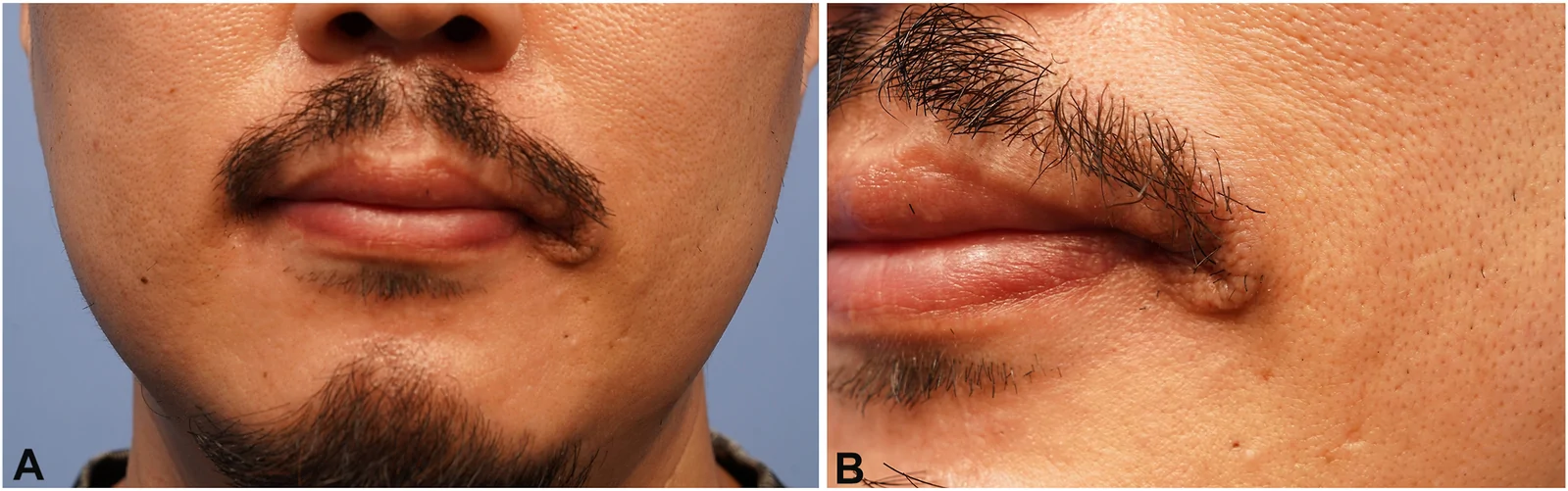
Sebaceous hyperplasia en plaque. **A,** A solitary, skin-colored to brownish plaque on the left perioral area creating the appearance of an accentuated unilateral perioral fold. **B,** Lateral close-up showing the smooth surface and preserved terminal hairs.

**Fig 3:**
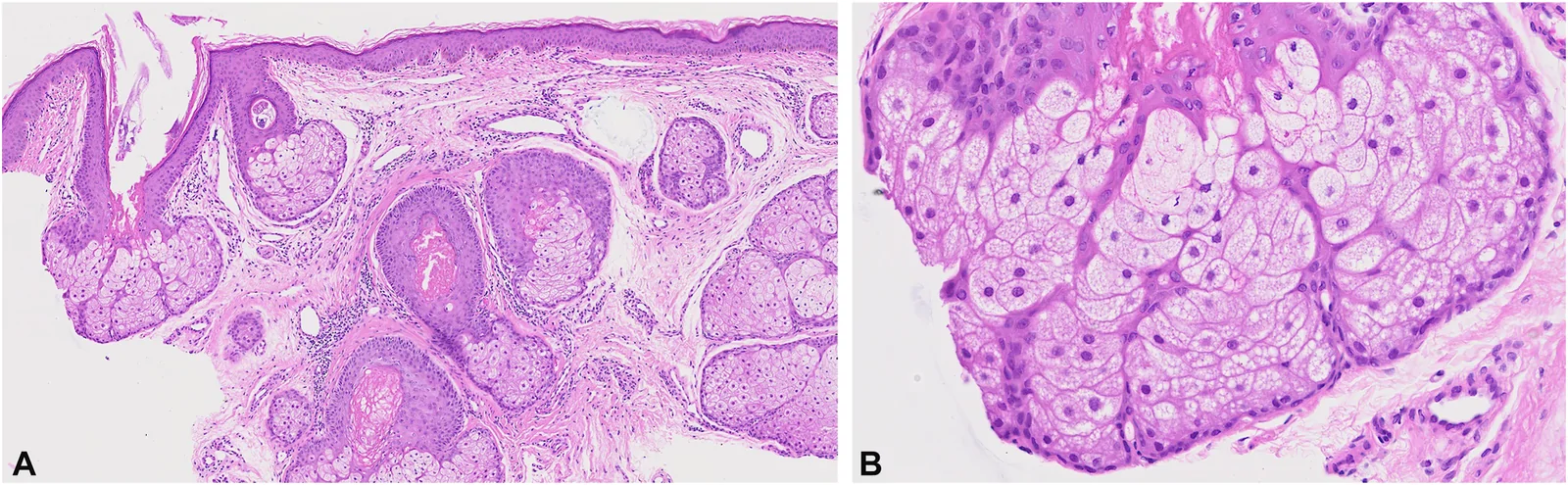
Sebaceous hyperplasia en plaque. **A,** Low-power view showing enlarged sebaceous lobules arranged around centrally dilated ducts, some opening onto the epidermal surface; the overlying epidermis is unremarkable without papillomatosis (hematoxylin-eosin stain, original magnification × 40). **B,** Higher-magnification view showing a lobule filled with mature sebocytes and a thin peripheral basaloid germinative rim limited to 1 to 2 cell layers, without cytologic atypia (hematoxylin-eosin stain, original magnification ×200).

**Fig 2:**
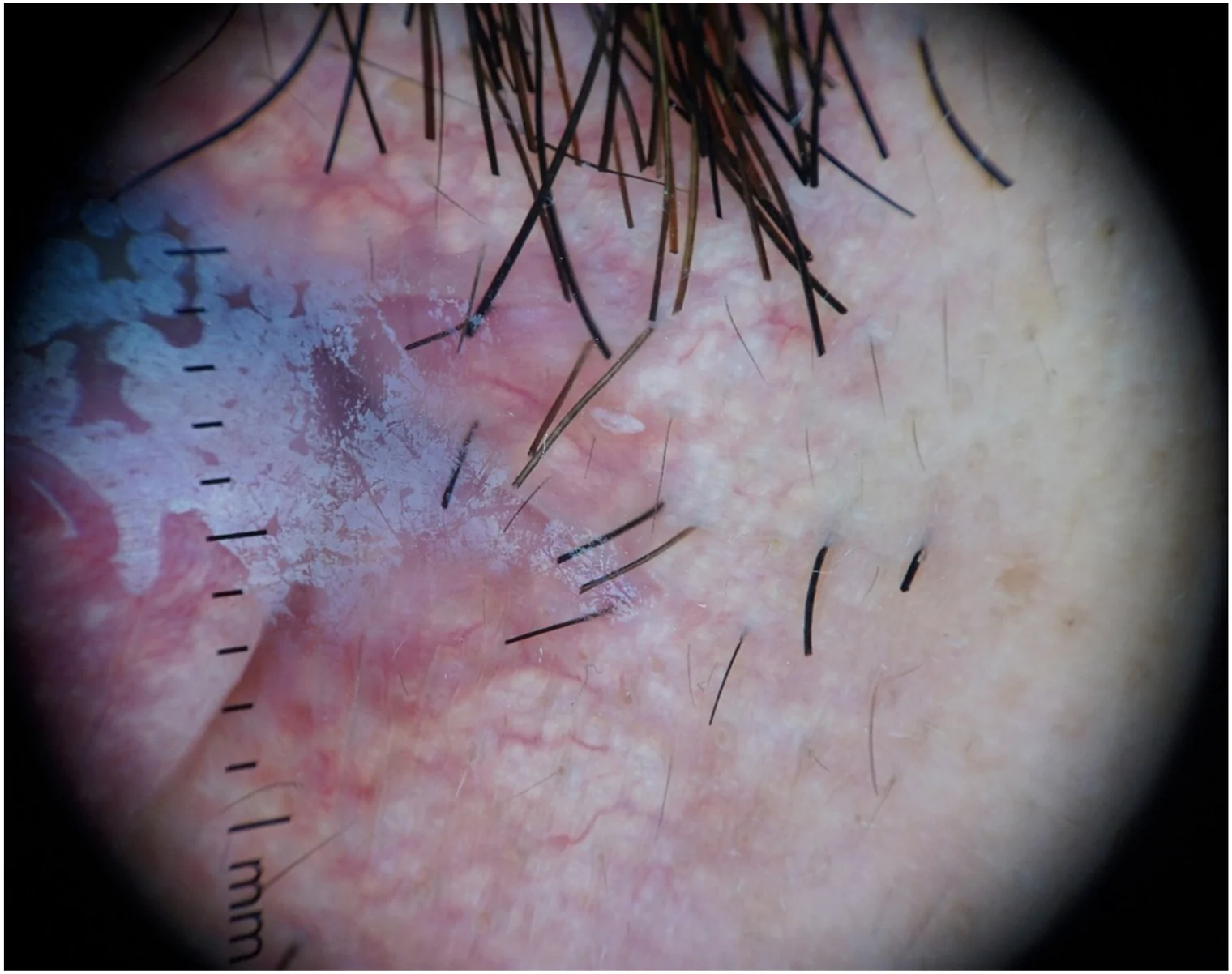
Sebaceous hyperplasia en plaque. Dermoscopy showing grouped whitish-yellow sebaceous lobules with peripheral crown vessels and preserved terminal hairs (polarized contact dermoscopy; ruler markings in millimeters).

**Question: What is the most likely diagnosis?**

**A.** Fordyce spots
**B.** Nevus sebaceous
**C.** Sebaceous adenoma
**D.** Sebaceous hyperplasia en plaque
**E.** Sebaceous trichofolliculoma

### Answer

**D.** Sebaceous hyperplasia en plaque.

## Discussion

Sebaceous hyperplasia (SH) is a benign proliferation of sebaceous glands that usually presents as multiple small, yellowish, umbilicated facial papules.^1^ SH en plaque is an uncommon variant in which the proliferation coalesces into a solitary, well-demarcated plaque. Reported cases are few and have involved varied head and neck sites, including the retroauricular area and cheek; similar lesions have also been described as giant sebaceous hyperplasia.^2^^,^^3^

The pitfall here was clinical. The lesion did not read as a tumor: its slow growth along a relaxed skin-tension line produced what resembled an accentuated unilateral perioral fold, and it was initially treated as inflammatory. Several features argue against a physiologic rhytid and warrant biopsy—the fold was strictly unilateral and solitary, well demarcated, slowly progressive, and studded with preserved terminal hairs. Dermoscopy showed grouped whitish-yellow lobules with peripheral crown vessels, supporting a benign sebaceous proliferation.

Sebaceous adenoma is a true neoplasm whose peripheral basaloid germinative population exceeds 2 cell layers within a disorganized architecture; here, the germinative rim was only 1 to 2 cells thick. Nevus sebaceous arises congenitally as an alopecic plaque with epidermal hyperplasia, papillomatosis, malformed follicles, and ectopic apocrine glands, none of which were seen. Sebaceous trichofolliculoma centers on a dilated primary follicle with radiating secondary follicles, which were absent. Fordyce spots typically present as multiple small yellow-white papules involving mucosal or mucocutaneous sites; in contrast, the vermilion border was spared in this patiens.^1^

Recognizing SH en plaque prevents both dismissing a persistent unilateral fold as physiologic and overcalling a bland proliferation as a neoplasm. SH is benign, and treatment is undertaken for cosmetic or functional reasons; options include destructive procedures, surgical removal, oral isotretinoin, and laser-based therapies.^4^^,^^5^ Surgical excision was performed in this patient. SH en plaque should be considered for a persistent solitary, smooth, unilateral facial plaque with preserved terminal hairs.

### Declaration of generative AI and AI-assisted technologies in the writing process

None.

## Conflicts of interest

None disclosed.
